# Real-World Performance of Conduction System Pacing Compared With Traditional Pacing

**DOI:** 10.1161/CIRCEP.123.012014

**Published:** 2023-06-19

**Authors:** Jordana Kron, Matthew Bernabei, Daniel Kaiser, Sudip Nanda, Faiz Ali Subzposh, Patrick Zimmerman, Rachael Rose, Kiah Butler, Kenneth A. Ellenbogen

**Affiliations:** Virginia Commonwealth University, Richmond, VA (J.K., K.A.E.).; Lancaster Heart and Vascular Institute, PA (M.B.).; St. Thomas Heart, Nashville, TN (D.K.).; St. Luke’s University Health Network, Bethlehem, PA (S.N.).; Geisinger Heart Institute, Wilkes-Barre, PA (F.A.S.).; Medtronic, Inc, Mounds View, MN (P.Z., R.R., K.B.).

**Keywords:** bradycardia, comparative study, database, electrophysiology, retrospective study

The deleterious effects of right ventricular pacing (RVP) are well known, including development of congestive heart failure and pacing-induced cardiomyopathy.^1^ Cardiac resynchronization can help improve clinical outcomes in patients with AV block and systolic dysfunction.^2^ Conduction system pacing including His bundle pacing (HBP) and left bundle branch area pacing (LBBAP) has emerged as an alternative pacing strategy to prevent or mitigate systolic heart failure.^3^ The purpose of our study was to compare the performance of leads in RV, HB, and LBBA positions in a real-world cohort.

We performed a retrospective cohort assessment to evaluate the performance of leads placed in the His bundle or left bundle branch area compared with right ventricular leads implanted on the septum or apex. The study included all patients implanted with a Medtronic pacemaker between January 1, 2020, and March 10, 2023, followed remotely on CareLink (Medtronic, Inc). This retrospective analysis of a deidentified data set was exempted from institutional review board review, and the requirement for informed consent was waived. Patients who received single- or dual-chamber pacemakers with Models 3830, 4076, or 5076 pacing leads in the ventricular position were included. Defibrillator and cardiac resynchronization devices were excluded. Pacing indication was recorded. Device data including pacing threshold (measured at a pulse width of 0.4 ms, as per the Carelink ventricular capture management algorithm), sensing, and impedances were captured. Inactivation of a lead, a surrogate for lead longevity, was defined as registration status inactivation (eg, explant or patient death) or the lead being programmed to off including change to AAI mode or output set to 0.5 V at 0.03 ms. Patients were censored at the time of their most recent transmission. Kaplan-Meier estimates of the cumulative probability of inactivation over time were calculated for each lead placement group, and a Cox model with a time-varying effect of lead placement group was fit. Statistical analysis was performed in R (version 4.2.1; R Core Team 2022). The analysis was performed with permissions granted through individual business associate agreements from each clinic site participating in the Carelink network. Restrictions in use of Carelink data, as governed by the business associate agreements, prevent sharing of data with other researchers.

We identified 224 814 total patients with an LBBAP lead (n=26 785; 11.9%), an HBP lead (n=8203; 3.6%), or an RVP lead (n=189 826; 84.4%). The most common implant indications for patients were AV block (14 057 [52%] LBBAP, 3970 [48%] HBP, and 75 609 [40%] RVP) and sinus node dysfunction (7646 [29%] LBBAP, 2788 [34%] HBP, and 87 010 [46%] RVP). The median R-wave amplitude at implant for LBBAP, HBP, and RVP was 13.00, 5.25, and 9.13 mV, respectively, and remained stable through 18 months of follow-up. The median pacing threshold for LBBAP, HBP and RVP at implant was 0.5V, 0.63V, and 0.5V respectively. From January 2020 through February 2023, the monthly percent of newly implanted leads in the HBP and RVP groups decreased while the monthly percent in LBBAP group increased to 31.9% (Figure). Eighteen months after implant, 13.0% of HBP patients had a threshold above 2V versus 1.8% of LBBAP patients and 0.7% of RVP patients (Figure). The lead inactivation rate at 3 years was 7.4% (95% CI, 6.8%–8.1%), 3.6% (95% CI, 3.1%–4.2%) and 3.2% (95% CI, 3.1%–3.4%) for HBP, LBBAP, and RVP patients, respectively (Figure). RVP patients had a lower lead inactivation rate than LBBAP patients during the first 30 days of implant (hazard ratio, 0.53; *P*<0.01) but a similar rate afterward (hazard ratio, 1.00; *P*=0.93).

**Figure.**
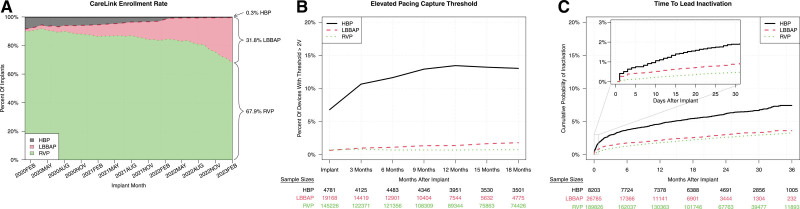
**Implant rate, pacing capture threshold, and time to inactivation rate for His bundle pacing (HBP), left bundle branch area pacing (LBBAP), and right ventricular pacing (RVP) groups. A**, CareLink enrollment rate by pacing method tracked from January 2020 to February 2023 for RVP (green), LBBAP (red), and HBP (black). **B**, Percentage of devices measuring pacing capture threshold >2 V from implant to 18 months for HBP (black), LBBAP (red), and RVP (green). **C**, Probability of lead inactivation for 3830 HBP (black), LBBAP (red), and RVP (green) leads over 3 years. Inset graph describes rates from 0 to 30 post-implant.

Conduction system pacing offers an alternative to RVP that can reduce the risk of pacing-induced cardiomyopathy and heart failure symptoms. While HBP demonstrates the safety and feasibility of true conduction system pacing, widespread adoption has been hindered by concerns for elevated and unstable pacing thresholds and small R waves.^4^ LBBAP has emerged as a new and improved conduction system pacing with low and stable pacing thresholds and R wave sensing typical of RVP.^5^ Our study shows that, in a real-world cohort of pacemaker patients followed remotely, the percentage of leads used for HBP decreased over time while the percentage used for LBBAP increased. LBBAP leads performed similarly to traditional RVP leads with low and stable pacing thresholds and longer time to inactivation compared with HBP leads. There are several limitations to this study including the observational post hoc design, lack of long-term follow-up, and lack of clinical outcomes in the CareLink database. Additionally, lead placement was determined from lead registration and not independently confirmed (with imaging or other approach).

The ideal pacing system will provide conduction system pacing that is identical to physiologic ventricular activation, with stable long-term lead performance and safe implantation technique easy for implanters to master. Our analysis of >220 000 leads demonstrates that LBBAP pacing is a step in the right direction, offering the best of HBP (safe, effective physiologic pacing) and traditional RVP (durable lead performance over time).

## ARTICLE INFORMATION

### Sources of Funding

This work was funded by Medtronic, Inc.

### Disclosures

Drs Bernabei and Subzposh have received honorarium from Medtronic. Dr Zimmerman, R. Rose, and Dr Butler are employees and shareholders of Medtronic. Dr Ellenbogen has received research support from Boston Scientific, Biosense Webster, Medtronic, St. Jude Medical, and the National Institutes of Health. Dr Ellenbogen is a consultant for Boston Scientific, St. Jude Medical, Atricure, and Medtronic. Dr Ellenbogen has received honoraria from Boston Scientific, Biotronik, Biosense Webster, Atricure, and Medtronic. The other authors report no conflicts.
